# Percoronary device occlusion of a large coronary artery fistula: Case report

**DOI:** 10.1097/MD.0000000000042830

**Published:** 2025-06-20

**Authors:** Run-Tian Pai, Zhen Tan, Li Hongxin, Shi-Bin Sun

**Affiliations:** a School of Clinical Medicine, Shandong Second Medical University, Weifang, Shandong, China; b Department of Cardiovascular Surgery, The First Affiliated Hospital of Shandong First Medical University & Shandong Provincial Qianfoshan Hospital, Shandong Engineering Research Center for Heart Transplant and Material, Jinan, China.

**Keywords:** coronary artery fistula, probe-assisted, sausage-like, transesophageal echocardiography, transthoracic occlusion

## Abstract

**Rationale:**

As a rare congenital heart malformation, coronary artery fistula (CAF) may lead to serious complications such as myocardial ischemia. While conservative management can alleviate symptoms, surgical ligation or interventional occlusion offers a definitive cure. This article reports a unique interventional case.

**Patient concerns:**

A 6-year-old female patient diagnosed as CAF presented with exertional chest pain, and imaging revealed a “sausage-like” aneurysmal dilation of the left anterior descending coronary artery, with a tail-like structure at its distal end draining into the right ventricle at an acute angle.

**Diagnoses:**

The patient was diagnosed with a CAF.

**Interventions and outcomes:**

The medical team innovatively employed a probe-assisted delivery system to successfully perform a transthoracic occlusion procedure under real-time guidance exclusively by transesophageal echocardiography.

**Lessons:**

This case demonstrates that, even in the face of complex anatomical pathways, this radiation-free, precise occlusion technique can achieve safe and effective therapeutic outcomes, providing a new and reliable option for the treatment of CAF.

## 1. Introduction

Coronary artery fistula (CAF) refers to an abnormal connection between the coronary artery and a cardiac chamber or another blood vessel, which allows blood to bypass the myocardial capillary bed and shunt directly.Particularly when the fistula is large, the affected coronary artery may gradually dilate over time, forming an aneurysm-like change. CAF may lead to serious complications such as pulmonary arterial hypertension, heart failure and myocardial ischemia etc.^[1]^ Hemodynamic significant CAF (in children) draining into the right cardiac chamber require timely definitive care to obviate cardiopulmonary complications. Conventional surgery and percutaneous techniques are the mainstay and approved approaches in the United States of America and Europe.^[2]^ However, percutaneous techniques may not be successful in highly tortuous and complex CAF courses. On the other hand, the application of conventional surgery in isolated and asymptomatic CAF remains a bone of contention.^[3]^

## 2. Case report

**Figure 1.**
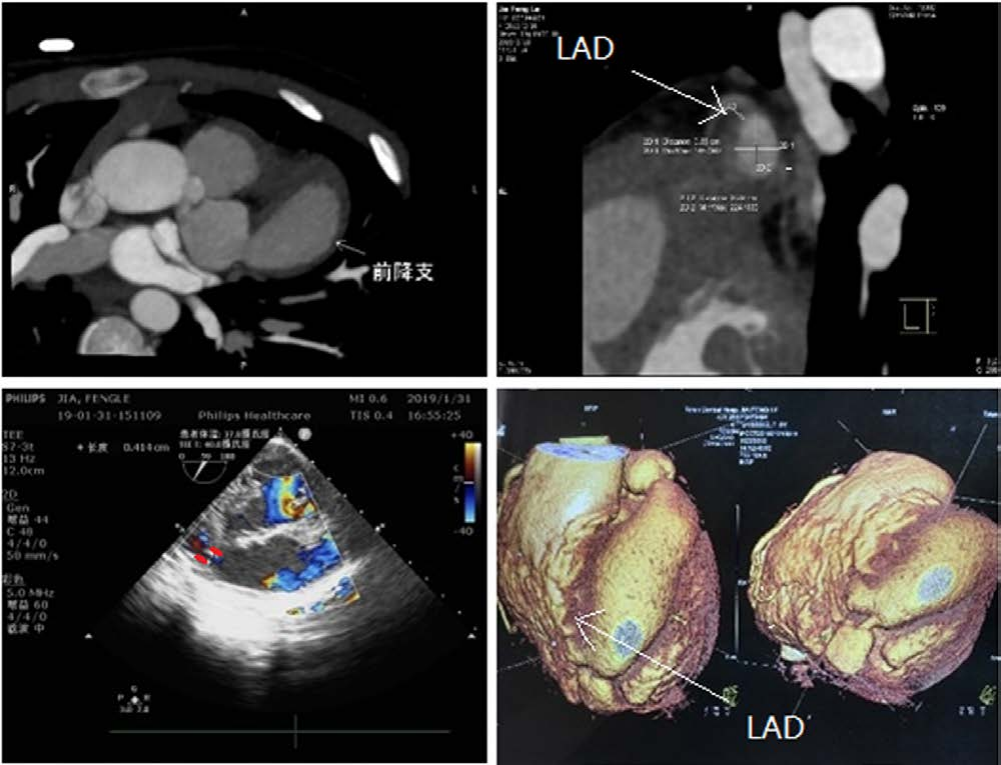
Sausage-shaped left anterior descending coronary artery fistula.

A 6-year-old female patient presented with exertional chest pain, and imaging revealed a “sausage-like” aneurysmal dilation of the left anterior descending (LAD) coronary artery, with a tail-like structure at its distal end draining into the right ventricle (RV) at an acute angle (Fig. 1 sausage-shaped LAD coronary artery fistula). Based on its complex anatomical pathway, we present an alternative and formidable technique of occluding complex CAF under exclusive TEE guidance.

## 3. Materials and methods

**Figure 2.**
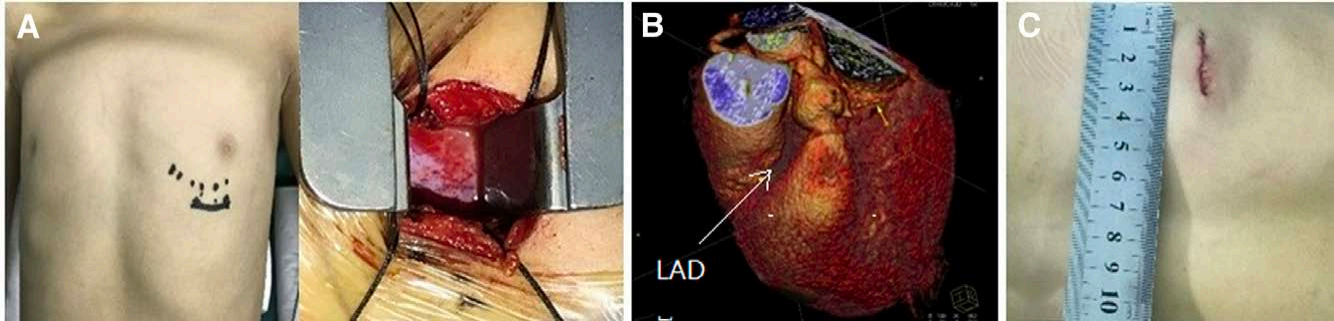
Surgical implementation (A) positioning of surgical incision. (B) LAD coronary artery embedded within the myocardium. (C) Surgical incision after closing. LAD = left anterior descending.

After standard general anesthesia and anticoagulation (heparin), in the supine position, a small incision (3–4 cm) is made in the left 3rd intercostal space, 1 and 2 cm from the sternum and below the nipple respectively (Fig. 2A positioning of surgical incision). Intercostal fascial and muscles are bluntly dissected, pericardium is accessed, incised, and cradled with 4 sutures. Despite having a large LAD, placement of purse-string suture was challenging, as the artery was embedded (bridged) in the myocardium (Fig. 2B LAD coronary artery embedded within the myocardium). Under TEE guidance, a search for a suitable place for purse-string suture placement was conducted by judiciously tapping on the distal LAD, while observing shunt response. Following a satisfactory search and purse-string suture placement, a puncture was made within the circle, and an appropriately sized sheath was introduced. Initially, attempts with a sheath and soft J-guide wire were made to crossover into the chamber, however, the angle was too acute for such a set.

After unsuccessful attempts with the aforementioned set, a metallic J-hollow probe was introduced to either crossover into the RV or align the orifice with the terminal LAD. The J-probe was utilized alongside soft J-guide wire, which was from time to time introduced into the hollow J-probe. Following the successful crossover of J-probe into RV, a guide wire was introduced and an F-6 sheath fed over it. An appropriate-sized occlude attached to a delivery cable with a stay-in suture was then deployed and later withdrawn following a satisfactory assessment of both the accuracy and stability of the device. The thoracotomy was closed in a standard fashion (Fig. 2C surgical incision after closing) without drainage and the patient was wheeled to the recovery room.

## 4. Results

**Figure 3.**
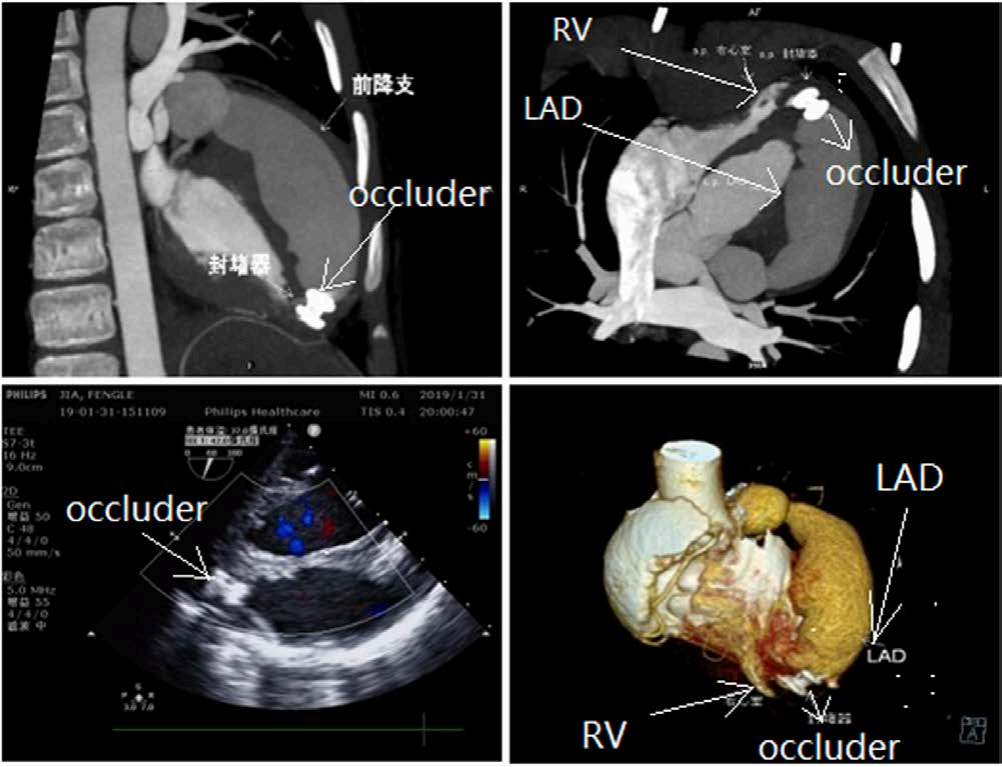
Imaging manifestations after occlusion.

Complete occlusion was achieved instantly after device release (Fig. 3 imaging manifestations after occlusion), with no conduction abnormalities. The patient was further managed according to a local team, as we were a visiting team. Since occlusion, no complication has been brought to our attention.

## 5. Discussion

As earlier alluded to, closure of CAF can be accomplished using open surgery (OS) ligation (endo or pericardial) or percutaneous occlusion (PO). With most cases having an asymptomatic course in children CAF if left untreated risk endocarditis and cardiopulmonary complications.^[4,5]^ Although the OS technique remains the gold standard in managing CAF,^[6,7]^ its application in an isolated and asymptomatic case such as ours is controversial.^[7]^ In addition, besides significant trauma and cardiopulmonary bypass-associated complications in the event of endocardial ligation, artery over-suturing in off-pump epicardial ligation has been reported. In 1983, Reidy^[6,8]^ and colleagues 1st reported PO of CAF using coils and detachable balloons, and since then other centers have followed. However, in 1 study^[8]^ of 17 cases, it was noted that only 5 were eligible for the PO technique. Besides femoral vessel challenges in infants and poor technical potency in severely tortuous CAF, PO has recorded several complications including coil embolization, T-wave inversion, etc. In 1 case, due to premature inflation of the balloon during the procedure, Reidy and his colleagues had to resort to OS. Given the anatomical complexity of our CAF, we feel, it mirrors the many failed cases using the PO technique.

## 6. Conclusion

Per-thoracic device occlusion of CAF using the probe-assisted delivery system under exclusive TEE guidance is a formidable alternative technique for managing CAF. The technique is efficacious and safe, regardless of coronary course complexity.

## Acknowledgments

We are highly indebted to the Director and Management of Tai’an Hospital for their trust in our capabilities and invitation to manage their patient.

## Author contributions

**Conceptualization:** Run-Tian Pai, Shi-Bin Sun, Li Hongxin.

**Data curation:** Run-Tian Pai, Shi-Bin Sun, Zhen Tan.

**Formal analysis:** Run-Tian Pai, Shi-Bin Sun, Li Hongxin

**Funding acquisition:** Zhen Tan, Li Hongxin.

**Investigation:** Run-Tian Pai, Shi-Bin Sun, Zhen Tan.

**Methodology:** Run-Tian Pai, Li Hongxin.

**Project administration:** Run-Tian Pai, Shi-Bin Sun.

**Resources:** Run-Tian Pai, Shi-Bin Sun, Li Hongxin.

**Software:** Shi-Bin Sun, Zhen Tan.

**Supervision:** Li Hongxin.

**Validation:** Run-Tian Pai, Shi-Bin Sun.

**Visualization:** Zhen Tan, Li Hongxin.

**Writing – original draft:** Run-Tian Pai.

**Writing – review & editing:** Run-Tian Pai.
